# Decentred regulation: The case of private healthcare in India

**DOI:** 10.1016/j.worlddev.2022.105889

**Published:** 2022-07

**Authors:** Benjamin M. Hunter, Susan F. Murray, Shweta Marathe, Indira Chakravarthi

**Affiliations:** a Department of International Development, University of Sussex, UK; b Department of International Development, King’s College London, UK; c SATHI, Pune, India

**Keywords:** Regulation, Accreditation, Insurance, Activism, Judicialisation, Corporate

## Abstract

In order to progress towards more equitable social welfare systems we need an improved understanding of regulation in social sectors such as health and education. However, research to date has tended to focus on roles for governments and professions, overlooking the broader range of regulatory systems that emerge in contexts of market-based provisioning and partial state regulation.

In this article we examine the regulation of private healthcare in India using an analytical approach informed by ‘decentred’ and ‘regulatory capitalism’ perspectives. We apply these ideas to qualitative data on private healthcare and its regulation in Maharashtra (review of press media, semi-structured interviews with 43 respondents, and three witness seminars), in order to describe the range of state and non-state actors involved in setting rules and norms in this context, whose interests are represented by these activities, and what problems arise.

We show an eclectic set of regulatory systems in operation. Government and statutory councils do perform limited and sporadic regulatory roles, typically organised around legislation, licensing and inspections, and often prompted by the judicial arm of the state. But a range of industry-level actors, private organisations and public insurers are involved too, promoting their own interests in the sector via the offices of regulatory capitalism: accreditation companies, insurers, platform operators and consumer courts. Rules and norms are extensive but diffuse. These are produced not just through laws, licensing and professional codes of conduct, but also through industry influence over standards, practices and market organisation, and through individualised attempts to negotiate exceptions and redressal.

Our findings demonstrate regulation in a marketised social sector to be partial, disjointed and decentred to multiple loci, actively representing differing interests. Greater understanding of the different actors and processes at play in such contexts can inform future progress towards universal systems for social welfare.

## Highlights

• The study of regulation in social sectors needs to recognise a wider range of state and non-state actors.
• Governments and statutory councils are important, but so too are accreditation companies, insurers, platform operators and consumer courts.
• Informal approaches based on individual and networked discretion also influence management and practice.
• Regulatory activities are performed in pursuit of actors’ own interests, with implications for social equity.
• Adopting decentred approaches to studying regulation can shed light on these complex systems and their implications for social welfare.

## 1 Introduction

Progress towards more equitable social welfare systems is likely to rely on an improved understanding of the forms that regulation takes in these sectors, and the implications for regulation of current transformations in the financing and provisioning of services. The regulation of corporate behaviour in primary and secondary industries has been an important area of discussion in development studies in light of the sustained expansion of commercial activities across borders and the formation of global value chains (Gereffi et al., 2005, Graham and Woods, 2006), and the mechanisms for ‘outsourcing governance’ that many states have pursued in response (Mayer and Phillips, 2017). Far less attention has been afforded to the regulation of commercial activities in social sector markets such as health and education (for recent exceptions from education see Baum et al., 2018, Härmä, 2019) – a gap that has become glaring since the onset of the SARS-CoV-2 pandemic and practices observed such as price gouging and refusals to provide treatment (Williams, 2020).

In this article we focus on the regulation of private healthcare. Private healthcare accounts for a significant minority, if not majority, of healthcare spending and visits to healthcare practitioners in many countries globally (Mackintosh et al., 2016). Growing attention to the heterogeneity of private healthcare sectors in many countries (Horton and Clark, 2016) has been accompanied by questions regarding the regulation of practices in this sector, and the role for public institutions in this (Montagu and Goodman, 2016). Considerable blame for regulatory failures has been placed at the door of governments who are felt to have lacked resources or failed to prioritise the regulation of private healthcare provision (Doherty, 2015, Hongoro, 2000, Sheikh et al., 2015), leading to a view that commercialised healthcare systems in settings such as South Africa and India are largely ‘unregulated’ (Choonara and Eyles, 2016, Contractor and Singh Kakar, 2020, Nandraj, 2012).

We offer an alternative perspective by adopting a definition of regulation used widely in public policy and regulation studies – as ‘the sustained and focused attempt to alter the behaviour of others according to defined standards and purposes with the intention of producing a broadly identified outcome or outcomes, which may involve mechanisms of standard-setting, information-gathering and behaviour modification’ (Black, 2002, p. 26). Informed by theory on decentred regulation and regulatory capitalism, we analyse data from a qualitative mixed-methods study conducted in the Indian state of Maharashtra to describe the eclectic set of regulatory systems produced by a broad range of formal and informal state and non-state institutions. Far from being unregulated, we find a sector where rules and norms are extensive but diffuse and reflecting the interests of a range of different actors; produced not just through laws, licensing and professional codes of conduct, but also through industry influence over standards, practices and market organisation, and through individualised attempts to negotiate exceptions and redressal. The state is conspicuous both in its sporadic presence as legislator and inspector, and in its reluctance to systematically regulate acts of provisioning. Here alternative systems have emerged that both reinforce and challenge the state’s role as regulator, in ways that are partial, disjointed and decentred, and which do not necessarily perform that regulation in the interests of public good.

The structure of the article is as follows. In the first section we outline what we see as an orientation in the study of healthcare regulation that has, to date, centred heavily on the activities of the state, and we point to important exceptions and a broader law, public policy and regulation literature that demonstrates the relevance of ‘decentred’ perspectives on regulation drawing attention to a range of actors and activities. We then describe the context for our empirical data and the case study approach used to generate and analyse our dataset. In subsequent sections we present findings from our analysis, and the article concludes with discussion on the key findings and areas for future study of decentred regulatory activities in social sector markets.

## 2 Healthcare regulation and its decentring

The regulation of healthcare provisioning is typically understood in terms of (dis)incentive structures offered by governments to promote and dissuade specific behaviours (Montagu and Goodman, 2016, Saltman, 2002). In most Anglophone countries and in the Anglophone global health and development literature this has been conceptualised as a hierarchy of ‘sticks’ and ‘carrots’ (Bennett et al., 1994) either administered directly by governmental organisations or through delegated authority to arms-length agencies and statutory professional bodies (Kumaranayake, 2000). Government agencies are expected to legislate, monitor adherence and administer warnings, fines and criminal proceedings against providers who fail to obey, while medical councils set and monitor standards, and censure those individual practitioners failing to perform appropriately (Dixon-Woods et al., 2011). To this end, state regulatory systems have incorporated a growing range of disciplining tools for audit and appraisal (Chamberlain, 2014).

Emphasis on state-administered regulation in existing literature reflects the historical basis for much of today’s allopathic healthcare: a biomedical model that arose in Europe, centred on a medical profession of physicians and their close entanglements with the modern state, and which subsequently expanded to other countries such as the USA (Freidson, 1988). Until the late 20th century, governments in these settings, and in newly independent states in Asia and Africa, afforded significant discretion and scope for self-regulation to the healthcare professions (notably medicine and nursing) on the basis that their unique knowledge and skills defied external control: the professions held responsibility for training and individual practice; and governments provided the statutory basis for this self-regulation and then focused on managing healthcare systems and organisations. While notions of professional self-regulation still hold sway in many settings, neoliberalisation processes since the 1980s have led many governments to draw back from direct involvement in healthcare management and provisioning, and to encourage the growth of large and diverse private healthcare sectors in their place, gradually shifting the locus of health work into private healthcare sectors. In this scenario governments were encouraged by the World Bank and other influential organisations to adopt purchaser-regulator functions as part of an idealised ‘regulatory state’ (Dubash and Morgan, 2012).

In practice, state regulation of private healthcare in many low- and middle-income settings is hamstrung by budgetary constraints and blurred boundaries between the public and private sectors. What has from a technocratic viewpoint been described as limited government ‘regulatory capacity’ (Ensor and Weinzierl, 2007, Montagu and Goodman, 2016), refers to the myriad problems facing government regulatory agencies: broad remits covering several sectors or areas of activity, limited statutory powers, inadequate funding, few (if any) dedicated staff, and issues of regulatory capture. They are undermined by the minimal incentives to design and adequately resource regulatory bodies amongst politicians who are deeply invested in the private healthcare sector, and amongst health professions reliant on income from fee-paying private users. Unsurprisingly, the agencies have failed to monitor and enforce adherence to ill-defined rules for a poorly understood private healthcare sector (Sheikh et al., 2015). In spite of interest from some scholars in how consumer organisations (Teerawattananon et al., 2003), and government-backed social health insurance programmes (Akhtar, 2011, Hort et al., 2013), might be used to regulate private healthcare, or in identifying opportunities to strengthen other institutions that can aggregate purchasing power in healthcare markets (Leonard et al., 2013), the regulatory role of the state and its agencies often remains limited when it comes to private healthcare. And what has arisen tends to be a set of alternative systems of control with varying degrees of direct and indirect involvement by state and non-state actors (Bloom et al., 2014, Montagu and Goodman, 2016).

India is a context in which discussion of healthcare regulation has tended to centre on the role of the state and professional licensing (Baru, 2013, Bhat, 1996a, Bhat, 1996b, Iyer and Jesani, 1999, Sriram et al., 2018, Sriram et al., 2020), with some attention devoted to the role of industry-led systems for voluntary accreditation (Chakravarthi, 2018), healthcare users and consumer law (Peters and Muraleedharan, 2008, Sheikh et al., 2013, 2015), and rights-based activism (Joshi, 2017, Shukla, 2018). With the design and enactment of regulatory systems now a key topic in discussions around the future of India’s healthcare system (Patel et al., 2021), there is a need for better understanding of the relevant actors and activities involved in regulating this sector.

Our contribution with this article is to bring into view a broader understanding of regulation, one which is used in some law, public policy and regulation literature, and in particular on ‘decentred’ perspectives to the analysis of regulation (Black, 2001) which permit consideration of a more expansive range of actors in setting the rules and norms of healthcare. In other sectors these actors have included: civil society (Hutter, 2006), credit ratings agencies (Scott, 2002), lenders of financial capital (Grabosky, 2013), and the now-ubiquitous insurers, auditors and management consultancies (Hutter, 2006). These modes of regulation have arisen in policy contexts of neoliberalism (Bartley, 2003) and where the ability of the state to regulate activities has been limited (Grabosky, 2013) and where legislative changes have encouraged adoption of regulatory roles by non-state actors (Braithwaite, 2006, van Rooij et al., 2016). Commentators point to a *decentring* of regulation from governmental ‘command and control’ mechanisms as regulation is instead ‘diffused throughout society’ (Black, 2001, p. 2): responsibility for different domains is given to, or taken by, a range of state and non-state actors, and there is growing variation in the instruments, controllers and controlees involved (Scott, 2004). This decentring of activities has been described as a shift from a ‘regulatory state’ (Majone, 1994) to a ‘post-regulatory state’ (Black, 2001) in a context of ‘regulatory capitalism’ (Levi-Faur, 2005) – a concept emphasising the interdependence of regulatory and capitalist institutions, such that ‘regulation made, nurtured and constrained the capitalist system and capitalism creates the demand for regulation’ (Levi-Faur, 2017, p. 289).

This decentred perspective on regulation has particular salience in healthcare, where 40 years of commercialisation has led to a pluralism of financing and provision (Mackintosh and Koivusalo, 2005), and indeed now regulation. Healthcare is, as Dixon-Woods (2019, p. 53) noted in the UK, ‘a new “polycentric” regime involving multiple agencies and actors that includes regulators, commissioners, insurers, academics, consultancy organisations, charities, and patients and their advocates.’ Work to document this regime been limited to specific modes of regulation in high-income settings, and there remain only cursory descriptions of the regulatory mechanisms beyond the state in the Global South, for example the activities performed by healthcare users and through media (Ensor and Weinzierl, 2007, Montagu and Goodman, 2016, Sheikh et al., 2013). We could not identify any work that has systematically applied a decentred approach to healthcare regulation in the Global South, though a small number of commentators have highlighted its potential utility in these contexts (Bloom et al., 2014, Hipgrave and Hort, 2014).

Studies conducted in Global North settings, by law, public policy and regulation scholars, indicate the potential for decentring the study of healthcare regulation. For example Jacobson (2001) outlined privatised systems of managed care and accreditation in the USA, while Trubek et al.’s (2008) special issue drew attention to a pluralism of institutions involved in the ‘regulatory ordering’ of contemporary healthcare, with a focus on healthcare in Europe and USA. Their interests tend to emphasise state roles, which is unsurprising given the prominence of state institutions in those settings, but they also note regulatory roles occupied by the ‘consumer/patient’ and a ‘plethora of private organizations’ (Trubek et al., 2008, p. 3), for example research governance roles for private employers, media and funding agencies (Rees, 2008). Healy (2017) similarly drew attention to the ‘patient as regulator’: a spectrum of activities encompassing the ways in which questions are asked of health professionals by users, and use of litigation to claim rights to safer care.

This article aims to add to the existing literature and discussions on healthcare regulation by applying a decentred approach to studying regulation in a middle-income context where market-based provisioning is widespread. We offer a detailed case study describing the range of state and non-state actors involved in setting rules and norms in this context, whose interests are represented by these activities, and the problems that arise.

## 3 Methods

### 3.1 Choice of case study

The site of our case study is Maharashtra state, India, which we selected because it is the country’s second-largest state by population and one of the most industrialised and urbanised (Government of India, 2011). Healthcare in the state has come to rely heavily on a private provider sector which is concentrated in urban areas (C. Chaudhari and Datta, 2020), as public healthcare has faced longstanding problems with under-resourcing (Radwan, 2005). But long-term trends for growth in private healthcare (Bhat, 1993) mask a shift in composition for what is a highly heterogenous sector. Historically, private healthcare in Maharashtra was dominated by individual private practitioners and small hospitals (up to around 30 beds), but the large private hospital segment has grown in recent decades (Nandraj et al., 2001), fuelled in part by an influx of private (domestic and foreign) investment (Hooda, 2015). Private hospitals in Maharashtra now outnumber those in the public sector by a ratio of more than three to one (2492 compared to 711 – Kapoor et al., 2020, p. 4), and although charitable hospitals occupy an important role within the private sector, particularly for low-income households (Marathe and Chakravarthi, 2019), the majority of the private sector operates on a for-profit basis. This care is largely paid for out-of-pocket by healthcare users (almost two-thirds of healthcare spending in the state is made out-of-pocket - Ministry of Health & Family Welfare, 2018), however there has been a push by federal and state governments to expand social health insurance schemes in recent years, and concurrent growth in employer and private health insurance coverage for workers with formal employment or higher incomes. The result is a recent, albeit small, decline in out-of-pocket expenditure nationally (World Bank, 2022).

The growth and evolution of private healthcare provision and spending in Maharashtra, and in India more broadly, has challenged a regulatory system dating back almost 100 years. British rule in India saw the institutionalisation of a European model for regulating healthcare practice that offers significant autonomy to leading professions through systems of licensing managed by statutory councils. Regional councils for medicine in India date back to the 1910s (Maharashtra Medical Council, 2015, Tamil Nadu Medical Council, 2020), and a national Medical Council of India was created in 1934, superseding the British General Medical Council’s role in the country (Jeffery, 1979). India’s independence in 1947, and the founding of its constitution, enshrined a federal system for governance in which jurisdiction for different sectors is divided between the federal government and the state governments; in the case of health, this is constitutionally the remit of the state governments. This encouraged the creation of further state-level statutory councils, including the Maharashtra Nursing Council, but also created opportunities for the introduction of legislation by state-level governments. The state governments of Bombay and Delhi each introduced a Nursing Home Registration Act requiring the registration of private healthcare facilities and periodic inspections (Bhat, 1996b). Specific clinical practices have also become the target of legislation at the state-level (for example Maharashtra, formed in 1960 from the bifurcation of Bombay state, introduced the Regulation of Pre-Natal Diagnostic Techniques Act) and at the federal level (for example Medical Termination of Pregnancy Act, Transplantation of Human Organs Act and Pre-Conception and Pre-Natal Diagnostic Techniques Act) (Government of India, 2007). The result is a mosaic of regulatory systems across the country which combine governmental legislation with professional self-regulation. As we will describe later in the article, in Maharashtra these systems have left substantial gaps and have been supplemented by a range of additional state and non-state actors from within and beyond healthcare.

## 4 Case study approach

In this article we present findings from case study research undertaken during 2017–2019 as part of a project aiming to document transformations in the provision and regulation of private healthcare in Maharashtra. We adopted a descriptive case study design (Yin, 2018) in order to analyse the decentred regulation of private healthcare in Maharashtra. For Yin, case studies can be categorised into three types: exploratory, explanatory and descriptive. The purpose of the latter being to present a complete description of a phenomenon within its context. Descriptive case studies have been used to study non-state regulation in a range of sectors and settings, including self-regulation amongst health professions (Butler et al., 2008, O’Meara et al., 2018) and amongst (non-health) companies and industries (Berkowitz and Souchaud, 2019, Jindra et al., 2019). As Marques notes in their article on the regulation of mining in Canada, the approach provides the opportunity ‘to observe the relationship between various forms of regulation as well as the interaction between different actors’ (Marques, 2016, p. 625). In our study we considered the private healthcare sector of Maharashtra as a single ‘case’ because of the importance of state-level legislation and professional bodies in regulating this sector. As indicated above, Maharashtra has been at the forefront of legislative developments to govern the private healthcare sector, and therefore represents a valuable case for close examination.

We used qualitative methods to collect data on issues relating to healthcare regulation. Semi-structured interviews were conducted with 43 respondents who have detailed knowledge of the private healthcare sector in this context, and we organised three witness seminars. A witness seminar is a specialised form of oral history group interview with ‘expert witnesses’ which has been used extensively by medical historians to document contemporary events (Tansey, n.d.). Our witness seminars addressed: transformation in the private healthcare sector in Maharashtra’s two largest cities – Mumbai and Pune – since 1980; and the introduction and implementation of specific government legislation to regulate healthcare. We supplemented interview and witness seminar data with a review of online press media. There has been a pronounced expansion in online press media in India, within a broader context of media commercialisation (Rao, 2010), and the reporting by local correspondents for national dailies as well as local newspapers provides an important source of information on state-level and local events, protests and court cases.

Potential interview and witness seminar respondents were identified through a purposive approach using online resources and professional networks, and were contacted by a member of the research team. They included leading clinicians from the cities, academics, activists and former state government officials. Respondents were informed about the aims of the research project, what participation would entail, and how data would be stored and used. Interview respondents were asked if the interview could be recorded and detailed notes were taken by a member of the research team in instances where permission for audio recording was withheld. Each witness seminar had 8–10 participants who had been involved in a particular event or series of events, and took place over a day. The witness seminars were audio recorded and edited transcripts were published after the participants had an opportunity to check the content. As is typical for witness seminars, participation in the seminars was contingent on consent for audio recording and the publication of transcripts as historical documents for public use; the published transcripts are available online (Chakravarthi and Hunter, 2019b, Chakravarthi and Hunter, 2019a) and are cited with page numbers at points in this article where we have used them to support the analysis. Ethics approval for the research was obtained from institutional ethics committees of Anusandhan Trust, Mumbai, and King’s College London.

The study findings reported in this article come from a framework analysis conducted on these data. This is a directed approach which uses policy- or theoretically informed questions to organise a dataset and allow closer interrogation. Framework analysis entails five steps: familiarisation, identification of a thematic framework, indexing, charting and interpretation (Pope et al., 2000). Our framework was informed by theory on decentred regulation and regulatory capitalism. It examined nine types of regulatory system through which state- and non-state actors produce and enact rules and norms for healthcare provision (Table 1). The findings are grouped in this article according to four themes which largely reflect the areas of analysis but with minor changes to aid narrative flow.

**Table 1:** Framework analysis and resultant themes.

Guiding areas for the framework analysis	Themes presented in the Findings section
Governmental regulation specifically targeted at healthcare	Weak state regulation
Other forms of governmental regulation that affect healthcare	State regulatory functions and their opposition
Professional self-regulation	Self-regulation and its limits
Regulatory activities performed by individual practitioners	
Activities performed by managers, investors and owners which regulate healthcare	
Self-regulation at an industry level	Decentred regulation, subdivided into:
Insurance regulation of healthcare	
Regulation by intermediary platforms that facilitate access to healthcare	• Partial self-regulation through commercial accreditation • Regulation through insurance • Regulation through marketplace platforms • User action and the judicialisation of regulation
Mechanisms through which users regulate healthcare	

## 5 Findings

### 5.1 Weak state regulation

State regulation of private healthcare provision in Maharashtra has been grossly inadequate for several decades, despite early recognition of its importance. At the time of independence, Maharashtra (then part of Bombay state) was one of the few Indian states where legislation was introduced relating to regulation of private healthcare provision: the 1949 Bombay Nursing Homes Registration Act (BNHRA). The Act was applicable to all of Maharashtra and allowed for local governments to introduce rules of implementation, but by the early 1990s it had only been adopted in the cities of Bombay (which would be formally renamed Mumbai in 1997), Pune, Nagpur and Solapur; in Bombay the municipal corporation only required that private healthcare facilities be owned by a medical doctor or qualified nurse who was considered fit to practice, and that the facility must be registered annually (Nandraj, 1994). In 2005 BNHRA was amended to provide a basis for government-set guidelines for nurse-patient ratio and floorspace-to-bed ratio that would be applicable in all districts in Maharashtra. This still left the Act limited in scope, even after a set of rules were published by the Maharashtra government in 2008 setting out expectations for providers to adhere to the Act (Chakravarthi and Hunter, 2019b).

The minimal requirements of the Act were poorly adhered to by both government and providers. Participants in our witness seminars explained how Maharashtra’s private healthcare sector grew rapidly from the 1970s onwards (Chakravarthi and Hunter, 2019a), and how instances of poor-quality care in the 1980s spurred public and activist interest in regulation (Chakravarthi and Hunter, 2019b). Investigations uncovered lapsed and missing registrations for private facilities, and by the late-1990s very little was still known in government about the private healthcare sector, as one former government official in the witness seminar noted: ‘in the Maharashtra State Assembly questions were raised about how many registered hospitals there are in Maharashtra. To our surprise we found that we had absolutely no idea’ (ibid., p. 23). Even now adherence is patchy: our own attempts to collect healthcare facility registration data were severely restricted as data were outdated and had not been categorised systematically.

Pressure mounted for the government in Maharashtra to take a more active role in the regulation of private healthcare. Initially this pressure came from activists and health users concerned with poor quality of care in the private sector. In 1990, the Bombay branch of activist network Medico Friend Circle, and Yasmeen Tavaria - the daughter of a deceased healthcare user, submitted a Public Interest Litigation case about private healthcare to the Bombay High Court after the user had been administered with an unmatched blood transfusion by a homeopathic doctor working in a private hospital (Chakravarthi and Hunter, 2019b, p. 15). The following year the court instructed the Maharashtra government to establish a permanent committee to oversee implementation of BNHRA and members of the committee went on to produce a report documenting the poor quality of care in Mumbai’s private healthcare system (Nandraj, 1992). Activists used this to continue to press the state government to adopt a set of rules to implement BNHRA, and to enforce adherence, throughout the 1990s and 2000s.

By the late-1990s, a recasting of government as regulator for private healthcare, rather than as a provider of its own services, increasingly aligned with the interests of national and international organisations, however the private healthcare sector itself appeared key in its opposition to state regulation of its activities. At the national level a series of federal governments (Bharatiya Janata Party-led National Democratic Alliance 1999–2004; India National Congress-led United Progressive Alliance, 2004–2014) were supportive of greater private provision of public healthcare services, while the World Bank’s USD 134 million Maharashtra Health Systems Development Project (1998–2005) included support for closer cooperation between public and private healthcare at the primary and tertiary levels. Nonetheless there remained a general reluctance amongst government and private providers to pursue the kinds of regulation envisaged by those wide-ranging interest groups. A former government official described the trepidation felt towards private healthcare regulation at the time: ‘The argument made by the private sector was that government hospitals were experiencing problems and we should first get our own house in order, before turning attention to others. For this reason we were not really pushing for standards in the private sector’ (Chakravarthi and Hunter, 2019b, p. 23).

The pivotal oppositional role occupied by private healthcare and its representations within professional bodies is illustrated in the Maharashtra government’s failure to adopt a Clinical Establishments Act (CEA), despite this being a 2010 federal Act (with accompanying rules published in 2012) that required adoption by individual state-level governments. There had been intensive lobbying by activists and non-governmental organisations through the Maharashtra chapter of the Jan Arogya Abhiyan [People’s Health Movement] for the adoption of the Act in Maharashtra, and the Maharashtra Minister for Health eventually set up an expert committee to draft the state-level bill. However the resulting bill disappeared into state government departments in 2015 and failed to re-emerge. From the start there had been national protests by doctors against the CEA led by the Indian Medical Association (IMA) on the grounds that systems of accreditation and existing laws could achieve the desired goals without giving government agencies such direct influence over the practices of private hospitals (Ekbal, 2012). Locally it was also being made clear ‘that the IMA is not happy. That was the main reason not to push forward. Indirectly, or directly, this feeling was that medical fraternity – the IMA – is not happy with this’ (former government official, in Chakravarthi and Hunter, 2019b, p. 49). Similar processes were playing out in other Indian states, notably Karnataka, as private medical lobbies proved adept at delaying and securing concessions from state governments who were attempting to introduce and implement their own version of the CEA (Shukla et al., 2021).

Renewed social movement impetus to adopt the CEA led to the formation of a second committee in 2018, again tasked with drafting a bill, however it appeared to be the drafting committee in name only. There was much disagreement between members, and the government official chairing the meetings frequently left early stating ‘discuss matters among yourselves, we will see later’ (drafting committee member, in ibid., p. 50). Then without consulting the committee the chair unexpectedly submitted a bill to the state government in 2018: ‘We don’t know what the final draft is like. I came to know that the Chairperson submitted the draft Bill to the government, but we don’t know what is included in it’. To clinician-activist interviewees and a witness seminar participant from the People’s Health Movement it was self-evident that the submitted bill will prioritise the interests of a segment of the medical professionals and the healthcare industry at the expense of user rights. It was an act of regulatory capture that reflected longer trends of professional obstruction to external regulation.

### 5.2 State regulatory functions and opposition to them

In contrast to the widespread user view that India’s private healthcare sector is being insufficiently regulated, especially on issues such as the cost and quality of care provided, a counter-narrative posed by some owner-clinicians and industry associations is that it is in fact *over*-regulated by the government and its agencies. Such claims are made on the basis that healthcare facilities have to comply with the healthcare-specific legislation for facility registration, pharmaceutical usage, and biomedical waste disposal, as well as more generic legislation relating to employment, building safety and company registration. For hospitals registered as charitable trusts there are required quotas for free or subsidised care to be made available to poorer users, and although these are to be monitored and enforced by the Maharashtra Charity Commissioner, interviewees reported that they are typically flouted.

While health is constitutionally the remit of state-level governments in India, there are some national rules that open new fronts for regulation, and opposition. One of the more recent examples is the National Pharmaceutical Pricing Authority’s (NPPA) price caps on hospital charges for cardiac stents and knee implants. The price cap on stents was brought about by a lawyer who, concerned with the large hospital bill a friend’s brother had received for receiving a stent, filed a Public Interest Litigation case in Delhi High Court in 2015 requesting stents be placed on the National List of Essential Medicines (Pawar, 2017). After several months of delay (and a second Public Interest Litigation case) the government implemented the court’s verdict and added stents to the list, then the NPPA introduced its price cap in early 2017. Later in that same year Prime Minister Narendra Modi announced that the federal government would reduce the cost of medical devices used in surgeries, and the NPPA, having recently concluded that healthcare providers were charging excessive mark-ups on knee implants, quickly placed a price cap on knee implants (Times of India, 2017b, Times of India, 2017). Critics – led by the Indian healthcare industry’s representative body NATHEALTH – variously allege that these price caps have led to use of lower quality products, slower development of new products, and even ‘reverse medical tourism’ from India to other countries, describing this as indicative of ‘regulatory overreach’ which threatens to ‘derail the robust growth of the sector’ (PwC and NATHEALTH, 2018, p. 2).

Criticism of existing and prospective regulation evokes historical concerns with government regulation in India. Before economic liberalisation, regulation in India’s economy was characterised by the ‘licence raj’ system of state licencing and quotas which governed who could produce and trade goods, and which was felt by some to undermine competition and to encourage corruption and bribery to secure permits (Mukherji, 2009). Critics have highlighted the emergence since the 1970s of an ‘inspector raj’ system that sees companies visited by representatives from government agencies and facing penalties, equipment seizures and closure for actual or perceived infringements of laws and policies (Das, 2006; The Indian Express, 2015), thereby creating further opportunities for corruption and bribery. Much of the existing systems for government regulation in healthcare rely on inspection formats that see facilities assessed according to their adherence to government rules, with the potential for fines to be levied in cases of non-adherence; and fears that further government regulation might extend an ‘inspector raj’ in healthcare were voiced during our witness seminar (clinician-activist, in Chakravarthi and Hunter, 2019b, p. 32). This resistance to forms of government regulation is indicative of an uneasy relationship between the medical profession and the regulation of its activities which, as noted in the previous section, has seen the IMA at the forefront of opposition to the CEA.

Divisions have opened up within the medical profession itself amidst concerns that larger hospitals have wielded disproportionate influence over policy-making through industry organisations such as Confederation of Indian Industry, Healthcare Federation of India, and Federation of Indian Chambers of Commerce and Industry (FICCI) Health Services Committee. This has challenged the influence of smaller and medium-sized providers who had previously been able to stymy attempts at state regulation. In 2011 the IMA launched the Hospital Board of India, which aims to ‘safeguard and help the interest of smaller hospitals’ (IMA Hospital Board of India, 2022), with the IMA President acknowledging the need to match the political influence of industry organisations and the corporate provider sector (Deccan Herald, 2011). The Nursing Homes Cell of the Association of Medical Consultants (AMC), an organisation established in 1978 to represent the interests of consultant physicians, was also involved in local lobbying against provisions in the proposed national CEA (Chakravarthi and Hunter, 2019b, pp. 40-46). The AMC was subsequently part of the 2014–15 drafting committee for the state-level CEA, but found itself out-manoeuvred by the larger providers (represented through the Confederation of Indian Industry) when the new committee was formed in 2018, and AMC’s position on the committee was only reinstated after protests to the committee chair (ibid. p. 48). These organisations represent a variety of models for healthcare provision, leading them to favour differing approaches to regulation and driving the fragmentation we document later in the article.

It is not just broad-brush legislation like the CEA that provokes resistance, but also more targeted legislation such as the Pre-Conception and Pre-Natal Diagnostics Techniques (PCPNDT) Act. The origins of the Act lie in the 1988 Maharashtra Regulation of Pre-Natal Diagnostic Techniques Act, which was introduced after campaigning by non-governmental organisations and activists on the issue of discriminatory abortions of female foetuses, and a subsequent national 1994 Pre-Natal Diagnostic Techniques (Regulation and Prevention of Misuse) Act which was amended to become the PCPNDT Act in 2003. The PCPNDT Act bans antenatal sex-determination, mandates registration of facilities offering antenatal diagnostic services and provides a set of rules including the public display of relevant information and the reporting of tests (using an ‘F-form’). A three-member committee – the ‘appropriate authority’ – comprising representatives from government health and legal departments and a non-governmental organisation such as a women’s rights organisation, is responsible for monitoring and enforcing the Act.

The inspections that take place under the remit of the PCPNDT Act fail to assess the quality of services being provided and instead fixate on procedural compliance in ways that permit claims that the government is active in this area. Inspections are motivated by public scrutiny of falling sex ratios, with government inspections and seizures of equipment closely following the publication of census data in 2001 and 2011 (Chakravarthi and Hunter, 2019b, p. 180), or following public scandals such as one involving a pair of doctors who were performing sex-selective abortions in the Maharashtra district of Beed (Dhupkar, 2019):.

‘I was witness to a video conference, conducted by Chief Secretary, and every Collector [the lead district-level civil servant] was there. The Chief Secretary asked, ‘How many sonography centres are there in your district?’ ‘220′. ‘So seal 20 centres’. Because they had to show that after the Munde case [in Beed] ‘we have taken action, we were so prompt’. So targets were given to the district authority: ‘There are 50 centres in your area, I want five centres sealed.’ (Head of a sonographic monitoring technology company, in Chakravarthi and Hunter, 2019b, p. 185)

Minor procedural infractions such as writing ‘NA’ rather than ‘Not Applicable’ on a form, or failing to display ‘Dr.’ on a nameplate in the clinic, are used as a basis for inspectors to suspend use of sonography machines and licenses for years pending appeal; and the lengthy process for High Court appeals means that the work of clinicians can be adversely affected for decades (ibid., p. 186). It is a superficial approach that fails to tackle the underlying causes of inappropriate and unethical practices, but which offers new opportunities for corruption and bribery. One witness seminar participant outlined the way in which PCPNDT Act inspections are used to extract payments from clinicians:.

‘Although Rs 25,000 is the official government fees for renewal of [PCPNDT] license, another Rs 25,000 is needed under the table. If you are a new applicant, namely for recent graduates, Rs 1 lakh [100,000] has to be paid under the table to get the registration under the Act, otherwise the authorities will not clear the papers for several months’ (radiologist, in Chakravarthi and Hunter, 2019b, p. 179).

The result is a regulatory system for private healthcare in which the state is both conspicuously present and absent: it makes detailed procedural demands of private providers that are enforced sporadically when it is politically (and financially) expedient to do so, but does little to assess the appropriateness of care being provided. For issues of clinical practice, much has instead been left to self-regulation amongst the health professions and in particular the medical profession.

### 5.3 Self-regulation and its limits

Statutory councils such as the Medical Council of India and the Indian Nursing Council have been key agencies for healthcare regulation in India through their control of education and licensing, administered through localised bodies such as the Maharashtra Medical Council (MMC) and Maharashtra Nursing Council, which are themselves underpinned by local legislation - the 1965 MMC Act and 1966 Maharashtra Nurses Act, respectively. Unlike voluntary members associations like the IMA, the professional councils have statutory powers for establishing and enforcing standards for clinical practice.

This self-regulation model has encountered several key problems in Maharashtra, and in India more widely. The first is porous boundaries around who is employed to provide what forms of care – known as ‘cross-pathy’. Much of the private healthcare in India - even in formal healthcare settings such as private hospitals – is provided by practitioners who have not been trained in allopathic medicine, but rather in alternative systems such as ayurveda and homeopathy. This was one issue in the Tavaria legal case mentioned in an earlier section, where a homeopathic doctor working in a private hospital had administered an unmatched blood transfusion. The employment of alternative practitioners to provide allopathic care serves several purposes: they are cheaper for hospital managers to employ than allopathic doctors, can fill staffing gaps in rural areas, and, in a recent national context of heightened Hindu nationalism, provide an opportunity to legitimise and deepen Hindu understandings of health and healing within biomedicine. However, alternative practitioners are not subject to the same professional oversight mechanisms as registered allopathic practitioners and there has been substantial resistance to ‘cross-pathy’ from professional associations such as the IMA who wish to protect their professional territory (Mint, 2018). The response of statutory councils such as the MMC has been somewhat weaker, indicating limits to their influence in this area, and has tended to centre on the issuance of a ‘show-cause’ notice to individual practitioners in response to complaints, requiring them to stop allopathic practice until they can justify their ability to do so.

The second problem is that medical self-regulation applies to individual practice and not to institutions. This is manifested in the case of advertising for services, which is forbidden by the MMC for individual practitioners as a breach of ethical guidelines, but is widely undertaken by larger hospitals without being subjected to the same restriction – something the IMA’s Hospital Board of India (representing smaller providers) has vocally opposed (Times of India, 2017a). The MMC has attempted to expand its jurisdiction to include regulation of private hospitals but has met resistance from healthcare companies. In 2014, for example, the MMC issued a directive – supported by the IMA (Times of India, 2014) – against cash-for-referrals (known as ‘cuts’ or ‘cut practice’) in which a referring health worker receives a ‘cut’ of the patient’s bill from the referred-to institution. The MMC instigated proceedings against two private hospitals accused of offering ‘cuts’ to doctors, however one of the hospitals then filed a case in the Bombay High Court to argue that the Council’s jurisdiction did not extend to companies. The Maharashtra government has since released details of its own draft bill to ban such ‘cuts’ (Barnagarwala, 2017), but it has yet to be heard in the legislature.

A third problem is that statutory bodies such as the MMC have failed to provide consistent leadership on issues of ethics. There are reports of the MMC failing to maintain its own membership registers in the 1990s (Bal, 1995), and a radiologist interviewee in Mumbai explained that the Medical Council of India’s code of ethics was not enforced by the MMC and is poorly understood by many recent graduates. There were problems with the MMC’s grievance redressal system too. A request filed under the Right to Information Act in 2015 showed that the MMC had 750 pending complaints at that time, of which 600 had yet to be examined and some had been pending for a decade. One healthcare user we interviewed described arduous attempts to seek redressal for a malpractice complaint with the MMC and its slow progress that took repeated visits, several years, and submission of detailed reports. Eventually the complainant was instructed that the case should have been filed against the surgeon and not the healthcare facility owner, and they have since resorted to consumer courts to seek redressal (see later section).

Maharashtra Medical Council itself is opaque and its functioning often contested in the state courts. A health activist described how in the 1980s bribery had become necessary just to get hold of documents setting out the governance systems for the MMC (Chakravarthi and Hunter, 2019b, p. 141). The Bombay High Court ruled against the MMC on multiple occasions in the 1990s, including in 1996 to insist users had the right to case papers relating to healthcare they received, and again in 1999 in response to concerns with vote-rigging in MMC elections. The latter ruling led to suspension of the MMC and impeded its functioning for another 12 years (Shelar, 2019). The MMC has increasingly become a site of contestation between the Maharashtra government and the medical profession, characterised by delays to elections and to taking up leadership positions, and accusations from MMC members that the Maharashtra government is trying to use its reserved appointments to the MMC’s ruling panel, and its influence over the choice of the Council’s registrar position, to legitimise and broaden cross-pathy practices (Dhupkar, 2016). These problems with the MMC reflect a wider national scenario of failed self-governance which was laid bare when the federal government dissolved the Medical Council of India in 2010, although it continued to operate until being replaced by the National Medical Council in 2019. This had followed the arrest of Council’s President on suspicion of corruption – taking payments in return for licensing private medical colleges (Pulla, 2014).

In this context responsibilities for professional self-regulation have been assumed by informal networks of practitioners, as well as more formalised groupings such as the Alliance of Doctors for Ethical Healthcare who articulate a vision of ethical practice and attempt to influence the practices of their colleagues. These networks have become visible in public campaigns to move away from systems of ‘cuts’ for patient referrals, which attracted significant attention in professional media (Gadre and Sardeshpande, 2017, Nagral and Nundy, 2017) and press media (Barnagarwala, 2017, Ravi, 2017). In 2017 a group of radiologists in Thane district, Maharashtra, shared an open letter pledging not to use ‘cuts’ (Shelar, 2017), and one private hospital placed an advertisement spurning ‘cuts’ and claiming to offer ‘Honest opinion. No commission to doctors’ (Times of India, 2017a). Doctors we interviewed stated that they personally did not ‘take cuts’ and would not refer a patient to a clinician or institution that did. However, even here there was acknowledged discretion in the ethical position with frank recognition that such a stance requires significant financial security and the local status to be able to continue to attract sufficient users and generate revenue without commercially rewarding referrals.

The failures of government regulation and professional self-regulation have resulted in a scenario for private healthcare of spiralling prices and growing concerns about unethical practices. Doctors we interviewed from small clinics and medical practices pointed out how investment in new facilities and technologies has become paramount for them to compete with larger corporate providers. But upgrading incurs substantial costs which must then be recouped through increased revenue from user fees, and this incentivises the use of unnecessary tests and treatments, and large mark-ups for services. Similar pressures to generate revenue permeate the larger corporate providers too. Opaque billing processes provide cover for these practices by downplaying the anticipated costs of services and masking the basis for final bills. It is a crisis that provoked the Bombay High Court to call in 2017 for greater transparency in hospital billings (K. Chaudhari, 2017), and which has driven a decentring of regulation as other actors attempt to control practices and pricing through their own rules, policies and norms. We turn to this set of alternative systems for regulation in the remainder of the article.

### 5.4 Decentred regulation

#### 5.4.1 Partial self-regulation through commercial accreditation

Accreditation systems have been seen by some actors as a way to set minimum standards of care at a facility level – distinguishing the ‘good’ from the ‘bad’ – without direct involvement from the government and its inspectors (see previous discussion on the CEA and PCPNDT) and without intervening in pricing and billing. Witness seminar respondents traced interest in private healthcare accreditation in Maharashtra back to the mid-1990s and growing concerns with practices in private healthcare sector (Chakravarthi and Hunter, 2019b, pp. 17-18). Multilateral and bilateral development organisations were encouraging interest within the Maharashtra government, for example funding Maharashtra government officials to travel abroad to study accreditation systems. Meanwhile, nongovernmental organisation CEHAT produced a set of minimum standards for healthcare and obtained funding from the World Health Organization to explore the potential for an accreditation system in collaboration with the AMC, whose leaders were concerned with disparities in quality and potential under-cutting in the sector:.

‘We were very keen [for accreditation] because many of us were unhappy with the standards which our members were following. It had been left to individuals, so I could improve my standards if I wanted to, but if my neighbour was not ready to do the same and his fees were half of mine then how would I manage?’ (former AMC President, in Chakravarthi and Hunter, 2019b, p. 18).

The result was two parallel attempts to produce local healthcare accreditation systems, led by state and non-state actors. The public process floundered, continuing a state intransigence outlined earlier in this article. Despite plans involving the Maharashtra Health Secretary and the Director of Health Services, the public accreditation system failed to materialise: ‘the government resolution remains in the file; two or three meetings took place and after that nothing happened’ (former state government official, in Chakravarthi and Hunter, 2019b, p. 24). The privatised system fared slightly better. Around 2001, the AMC launched a Healthcare Accreditation Council but it proved unable to enforce standards for care and resorted to finding a partner to manage the system. One proposal from an Indian credit rating agency was rejected as membership fees would be too high, and eventually a newly formed not-for-profit led by an engineer – the Forum for Enhancement of Quality in Healthcare (FEQH) – took on the accreditation; it has now has certified 350 facilities, which are predominantly small clinics and hospitals based in Mumbai (Chakravarthi and Hunter, 2019b, p. 18).

At a national level there were more sustained attempts to develop an accreditation system by an alliance of government and industry organisations, culminating in the 2006 launch of the National Accreditation Board of Hospitals and Healthcare Providers (NABH) under the management of the quasi-governmental Quality Council of India. The composition of NABH’s governance structures gives an indication of its role in promoting industry interests for healthcare as its permanent founding members are FICCI, the Confederation of Indian Industry, Associated Chambers of Commerce and Industry of India, Ministry of Consumer Affairs, and Council of Scientific and Industrial Research (Quality Council of India, 2019, p. 52). The Ministry of Health and Family Welfare and professional associations are notable absentees and fall instead within a group of other government ministries and agencies, and private healthcare organisations, who are represented on the NABH board. The development of NABH reflected an industry-led self-interest to address domestic and international concerns with quality of care that threatened to undermine India’s emerging position as a global medical travel destination. The expansion of NABH to include other areas of healthcare that are popular with medical travellers, such as dentistry and alternative therapies, as well as to include other services, such as those offered by medical travel facilitation agencies, underlines this association between NABH accreditation and global healthcare markets.

Seeking to regain influence within this emerging regulatory system, the IMA’s Hospital Board of India signed an agreement with NABH in 2015. Through this agreement the IMA would begin promoting NABH accreditation to its smaller and medium size hospital members (Rana, 2015), buttressing this quasi-governmental regulatory system while undermining the case for a CEA, which the IMA had already opposed. However, at the time of writing the NABH website reports that in Mumbai there are 21 allopathic clinics and 25 hospitals accredited by NABH, and in Pune there is one registered clinic and 23 hospitals (NABH, 2021). These reflect only a portion of private healthcare provision in these settings, and are typically facilities that are larger and interested in markets for the insured middle-class and for global healthcare users.

#### 5.4.2 Regulation through insurance

The insurance industry has been motivated to intervene to address issues such as price inflation and unnecessary testing and treatment in private healthcare. As one of our witness seminar participants noted, a dual movement is taking place: ‘insurance is going to control the charges and NABH is going to control the standards’ (former AMC President, in Chakravarthi and Hunter, 2019b, p. 52). The World Bank has supported efforts to bring insurance companies together with NABH to produce a set of standards for healthcare providers in India (Smits et al., 2014), and the federal government has been supportive of this shift, for example proposing that NABH-accredited hospitals should receive reimbursements that are 10–15% higher than non-accredited hospitals through the national social health insurance scheme, Ayushman Bharat (Quality Council of India, 2019).

Historically health insurance has been concentrated amongst government workers through the federal government’s Central Government Health Scheme, subsidising their purchase of healthcare services in private hospitals. In recent decades, however, the growth of the urban middle-class, and the companies they work for, has encouraged public and private insurance companies to expand the health insurance market with new products aimed at individuals and employers. State-level and federal governments have formed their own publicly funded health insurance schemes, managed through autonomous trusts and insurance companies, to cater to low-income and informal sector workers. As health insurance continues to grow – currently markets are increasing by 20% per year (Invest India, 2021) – these companies are gaining more and more influence as collective purchasers of services.

In a bold public statement entering this regulatory territory in 2017, shortly after Prime Minister Modi announced that a new law was to be brought in requiring doctors to prescribe medicines by their generic names, one private insurer – Max Bupa Health Insurance – took the matter into its own hands. They wrote to inform hospitals that they must prescribe medicines only with their generic names to reimburse claims, and sought compliance with ‘immediate’ effect, adding that a clear justification of not using generic drugs under certain circumstances would have to be written and filed as a part of discharge documentation. Max also warned hospitals that it has the right to retrospectively audit those claims where there are no justifications given for prescription of branded drugs. The IMA’s Hospital Board of India fought back by appealing to the notion of professional autonomy on the grounds that ‘intruding into the right of registered medical practitioners from prescribing quality drugs for his patients is unwarranted and unethical’ (Sinha and Rajagopal, 2017).

In Maharashtra, there is a mix of private and public insurance companies but four publicly owned companies provide cover to the greatest portion of the insured population (estimated to be around three-quarters by one of the witness seminar participants). They are represented collectively through the General Insurance Public Sector Association (GIPSA), which provides a collective bargaining body for empanelling hospitals and which has attracted criticism from the hospital industry for its attempts to restrict much-desired (and more equitable) ‘cashless’ policies to a subset of hospitals that accept discounted rates: its Preferred Provider Network (GIPSA PPN). Although that restriction was later relaxed, it is emblematic of the bargaining power held by GIPSA and the extent to which it can regulate healthcare pricing by compelling hospitals to follow its policies.

Regulation by private and public insurance companies has penetrated deep into private hospitals’ clinical departments. Insurance companies have established their own clinical pathways through standardised treatment protocol that set out the tests and treatments to be provided in the event of particular health conditions. These protocols are used to standardise care and enable the capping of fees that will be paid by an insurer, calculated according to a negotiated cost for the package of services. Medical care has to be documented in ways that can be audited by administrators and by the clinicians employed by insurers and third-party administrators (TPA) to review claims. Insurers determine the forms of care that are deemed appropriate to be covered by the insurance, and the items that are considered additional for which users must settle the bill.

In this system, insurance companies operate both as regulators and regulatees (Schmidt and Scott, 2021). The use of TPAs by insurance companies, as part of their processes for verifying the necessity of treatments provided and negotiating reimbursements with hospitals, aroused criticism from practitioners and owners as these agencies attempt to aggressively renegotiate reimbursements to hospitals: ‘the insurance company and TPA are hell bent upon giving you about 40 to 50% less than what the normal charges are’ (senior AMC leader, in Chakravarthi and Hunter, 2019b, p. 34). In a reflection of wider reliance on judicial intervention to which we return later, it took Public Interest Litigation filed in 2011 by an activist, and resulting intervention by Bombay High Court in 2015, to press the Insurance Regulatory and Development Authority of India (IRDAI) into action regarding the behaviour of TPAs. The IRDAI then produced guidelines relating to the activities of insurance companies and agents acting on their behalf (Bhasin, 2015, Kothari, 2015). Users have also resorted to the relatively unknown insurance ombudsman to seek redressal for complaints against the insurance companies (Johari, 2016).

#### 5.4.3 Regulation through marketplace platforms

Technology companies are now seeking to make similar inroads into private healthcare markets and are producing new tools for the regulation of healthcare practices. In the past 10–15 years several digital marketplace platform companies have expanded in India with a view to positioning their platforms as intermediaries for accessing healthcare. Platforms such as Practo, Lybrate and PSTakeCare offer listings of healthcare providers accompanied by locations, prices and ratings, with the opportunity to filter and book services. Often founded by technology entrepreneurs, and with backing from global investors, they use commercial models copied from platforms in other sectors and seek to generate revenue through subscriptions, one-off fees, or one-off fees charged as a proportion of medical bills. New services have been introduced which create new needs for providers and users to engage with platforms, for example through introduction of video consultations and practice management software. A clinician interviewee used one marketplace platform’s cloud storage system to manage their users’ medical records; another said they knew clinicians who used platforms more like appointment management systems.

Marketplace platforms increasingly emulate insurers as gatekeepers to healthcare. Each platform seeks to create its own bounded virtual marketplace in which users can browse, filter and book. Platform moderators can restrict participation to particular providers and users – those who are willing to accede to membership rules regarding behaviour and data usage. Multiple clinician interviewees noted being approached by representatives from platform companies that wanted to list them on their platform, to expand the marketplace and options available to users. Some described being offered priority positioning in the platform’s online listings, if they paid a fee, but also noted that this might compromise medical council restrictions on advertising by individual clinicians. In a claim that is revealing of the pressure on platforms to expand quickly, interviewees reported being listed on a platform without their prior knowledge and consent. This raises questions about the conduct of a platform sector that itself falls within a regulatory grey area, as neither provider nor purchaser of healthcare services, and which lacks the same kind of dedicated regulatory agency and ombudsman that exists for insurance.

User reviews posted on provider profiles in marketplace platforms pose a particularly acute mechanism for encouraging and discouraging some practices by providing a public forum for critical feedback and the airing of grievances. Comments influence how prospective users perceive a provider, in turn affecting demand for that provider’s services. Providers are therefore heavily incentivised to pursue practices that will protect and enhance their digital reputation: in much the same way that litigation in healthcare has driven ‘defensive practices’ in medicine (see next section), the use of review systems in marketplace platforms is liable to engender ‘digi-reputational practices’ that anticipate and respond to the concerns of the platform consumer. It is an area of regulatory activity that is intensified by listing algorithms that factor user comments and feedback into the order of providers that appear in user searches (see for example Practo, 2021), prioritising those providers whose profiles report better user satisfaction. These activities point not just to the growth of industry actors in regulating healthcare practices, but also to the role of individual users in performing regulatory activities.

#### 5.4.4 User action and the judicialisation of regulation

Healthcare users have pursued a range of informal and formal mechanisms to influence norms in healthcare and to prompt government and other agencies to take action in this area. The demands placed on the market by middle-class consumers desiring hotel-like facilities, advanced technologies, and on-site specialist and emergency care, are felt to have played some part in driving price inflation and unethical practices. Users then frustrated with unsatisfactory outcomes in private (and public) healthcare facilities resort to seeking various forms of justice. Disgruntled users and family members take to social or press media to share grievances and our respondents repeatedly pointed to an example that received national attention after a media campaign instigated by the parent of a deceased child who complained that they had been overcharged by Fortis in Gurugram, sparking a state government investigation, referral to the Medical Council of India and a police case (Krishnan, 2019). Attacks on workers in healthcare settings have also become a concern, in spite of targeted legislation – the 2010 Maharashtra Medicare Service Persons and Medicare Service Institutions (Prevention of Violence and Damage of Property) Act – and a 2014 Bombay High Court ruling that requested its implementation (Saigal, 2017). In June 2019 doctors in Maharashtra’s public and private hospitals went on strike as part of a national protest against the violence. As a participant in the witness seminar noted, violence is an issue ‘that is in everybody’s mind’ (Chakravarthi and Hunter, 2019b, p. 32).

While facilities respond to the airing of grievances through their own social media accounts and libel threats, and to physical threats of violence with an enhanced security presence, it is the threat of consumer litigation that appears most compelling and which augments the regulatory role of the state’s judicial arm, mirroring trends for judicial intervention in the healthcare systems of other countries (Lamprea, 2017). Public Interest Litigation has been used repeatedly to press government agencies to regulate healthcare provision and financing in Maharashtra, including several cases mentioned throughout this article, however the application of the 1986 Consumer Protection Act (and its successor 2019 Consumer Protection Act) to healthcare provision is the spectre that haunts private healthcare providers. Two legal judgements in Kerala in 1992 brought private healthcare within the purview of the Consumer Protection Act: one seeking compensation for the death of a user in Cosmopolitan Hospital, Thiruvananthapuram; the other regarding the loss of uterus for a user of Lakshmi Hospital, Cochin. The outcome of the national appeals relating to these cases was that users of private healthcare services would be able to lodge complaints to a district-level forum on the grounds of receiving deficient care (Bhat, 1996a).

The potential for litigation has driven new practices on both sides of the ‘provider-consumer’ relationship. For hospitals and practitioners, there is growing incentive to over-test and over-intervene, as part of a broader trend towards precautionary and ‘defensive practice’ which has been the subject of debate elsewhere (Saha and Shetty, 2014), further intensifying the inflationary processes we described earlier. Our respondents described other mechanisms for legal protection in clinical decision-making, such as disclaimers and consent forms, with one clinic going as far as to video-record the medical consent process to ensure evidence of user agreement. As well as assisting compliance with the regulatory apparatus of the insurance industry, the introduction of standardised protocol in healthcare chains and facilities speaks to the concern with providing and documenting care in ways that will be admissible in court proceedings.

In turn some healthcare users – now designated as ‘consumers’ – anticipate a need for litigation and prepare accordingly. One such interviewee described a methodical process they used to document and monitor care by requesting copies of their itemised bills on a daily basis. Another, who was pursuing litigation against a small private hospital, used blogposts and a Facebook group to detail and share their experiences with legal proceedings publicly. A Whatsapp group they set up with other healthcare users turned into a patients’ forum through which people could share tips on the legal process.

The extent to which healthcare in Maharashtra has now been drawn into the realm of individual consumer rights as a basis for judicialised regulation is demonstrated through the growing attention given to a proposed Charter for Patients’ Rights in India. After decades of advocacy by non-governmental organisations, and building on a list of rights prepared by the National Human Rights Commission, in 2018 the federal government’s Ministry of Health and Family Welfare launched a consultation on a proposed Charter of Patients’ Rights that encompasses 17 rights, including rights to: second opinion; transparency in rates; alternative treatment options if available; choose the source for obtaining medicines and tests, and ‘to be heard and seek redressal’. The 1986 Consumer Protection Act featured prominently in the consultation proposal as providing the basis for many of the rights included (National Human Rights Commission, India, 2018), and while the hope amongst some proponents appears to be that this will encourage greater accountability in private healthcare provision, it also emphasises individual rights and claims, and potentially side-lines considerations of social equity.

## 6 Conclusion

In the study of development, the regulation of commercial activities in social sector markets such as health has typically focused on state legislation and the transfer of governance functions to self-regulating professions. In settings and sectors where legislation is partial, rules poorly enforced, and professional self-regulation undermined, this ‘regulatory state’ perspective encourages claims that activities are ‘unregulated’. By adopting a definition of regulation that is more commonly used in other areas of public policy and regulation studies, we have argued that there is a broader range of actors who perform regulatory activities in order to promote the interests of their own constituencies. Their activities are better understood as part of a wider scenario of regulatory capitalism in which regulation is not absent but is partial, disjointed and decentred to multiple loci.

In this article we have outlined several regulatory systems that coexist and operate in a middle-income context where market-based provisioning is extensive. In Maharashtra’s private healthcare sector, we have pointed to centres of regulation that are based not in the corridors of health ministries and statutory professional bodies as is often traditionally assumed in the global health and development literature, but rather are situated in the offices of regulatory capitalism: accreditation companies, insurers, platform operators and consumer courts. The government and statutory councils perform some limited and sporadic regulatory roles, typically organised around legislation, licensing and inspections, and often prompted by the judicial arm of the state, but a range of industry-level actors, private organisations and public insurers are involved too, in order to promote their own interests in the sector. Their fragmented attempts to control practices and billing, and to distinguish ‘good’ from ‘bad’, are motivated by a need to protect their own status and bottom line, and often sit in tension with regulatory approaches that are instead grounded in notions of social equity and accountability (Shukla et al., 2018). It is noticeable that where progress has been made to develop and implement government regulation, this has come about only after substantial effort on the part of healthcare users, their family members, and networks of professionals and activists. This can be, and is, used as inspiration for securing better and fairer access to healthcare in countries around the world through global networks such as the People’s Health Movement and the Community of Practitioners on Accountability and Social Action in Health.

Our research points to the importance of recognising the informal regulatory activities that govern behaviours. In this we have drawn from the example of Scott (2004) and others who have studied public regulation in high-income Anglophone settings, where informal practices have been central to self-regulation amongst medics (Waring, 2007, Waring et al., 2010), as social norms and varied interpretation of guidelines act to shape the clinical behaviours of individual practitioners (Kilminster et al., 2010). In the case of Maharashtra, codified rule systems for managing and standardising clinical care, insurance financing and platform-based interactions run alongside informal approaches based on individual and networked discretion. It is a regulatory system that combines US-style corporatisation and managerialism with a social environment characterised by significant scope for individual discretion and exception. Unfortunately, this system appears to largely support the interests of institutions and individuals who have sufficient financial and social capital to navigate those hospital, insurance, and legal bureaucracies.

This initial step towards understanding the range and function of different actors in the regulation of social sector markets encourages more detailed examination of regulatory activities and the connections between them: Who performs regulation and what are the incentive structures that they create? Whose interests are represented and promoted by these actors? And, in a context where several groups of actors are both regulators and regulatees, who should regulate the ‘regulators’? Addressing these questions in Maharashtra state, in India, and in other middle-income countries, can inform the progress towards achievement of universal and more equitable social welfare systems.

## CRediT authorship contribution statement

**Benjamin M. Hunter:** Conceptualization, Investigation, Formal analysis, Writing – original draft. **Susan F. Murray:** Conceptualization, Methodology, Writing – review & editing, Supervision, Project administration. **Shweta Marathe:** Investigation, Writing – review & editing. **Indira Chakravarthi:** Conceptualization, Methodology, Investigation, Writing – review & editing, Supervision.

## Declaration of Competing Interest

The authors declare that they have no known competing financial interests or personal relationships that could have appeared to influence the work reported in this paper.
